# Salvage endoscopic ultrasound-guided gastrojejunostomy as a bridge to definitive surgical therapy for duodenal adenocarcinoma presenting with duodenal stent obstruction

**DOI:** 10.1007/s12328-023-01781-2

**Published:** 2023-04-08

**Authors:** Tiffany Z. Yu, Abishek Agnihotri, Richard Zheng, Babar Bashir, Nayeem Nasher, Charles J. Yeo, Avinoam Nevler, Harish Lavu, Wilbur B. Bowne, Anand Kumar

**Affiliations:** 1 Sidney Kimmel Medical College, Thomas Jefferson University, 1025 Walnut St, Philadelphia, PA 19107 USA; 2 Bayhealth Medical Center, Dover, DE USA; 3 Department of Surgery, Johns Hopkins Hospital, Baltimore, USA; 4 Department of Medical Oncology, Thomas Jefferson University Hospital, Philadelphia, USA; 5 Department of Surgery, Thomas Jefferson University Hospital, Philadelphia, USA; 6 Department of Gastroenterology and Hepatology, Thomas Jefferson University Hospital, Philadelphia, USA

**Keywords:** Gastrojejunostomy, Endoscopic ultrasound, Gastric outlet obstruction, Lumen-apposing metal stent, Whipple procedure

## Abstract

The utilization of endoscopic ultrasound-guided gastrojejunostomy (EUS-GJ) in the setting of an obstructed (ingrown) duodenal stent as a bridge to pancreaticoduodenectomy (PD) remains undescribed. Herein, we report a case study of a 51-year-old patient who underwent EUS-GJ using lumen apposing metal stent (LAMS) for an obstructed duodenal stent during neoadjuvant treatment for duodenal adenocarcinoma. The patient ultimately underwent surgical resection by a classic PD 14 weeks after LAMS placement. EUS-GJ using LAMS represents a potential option as a salvage bridge to surgery for duodenal obstruction in the setting of an obstructed duodenal stent.

## Introduction

Duodenal adenocarcinoma (DAC) is a rare gastrointestinal (GI) tumor that may cause gastric outlet obstruction necessitating placement of a duodenal stent or surgical bypass. Advances in therapeutic endoscopy now include endoscopic ultrasound-guided gastrojejunostomy (EUS-GJ) using a lumen apposing metal stent (LAMS) which adjoins and anchors two GI lumens together [1]. In the palliative setting, EUS-GJ demonstrates promising clinical efficacy compared to the traditional duodenal stent and/or surgical gastrojejunostomy (GJ) [2]. Currently, EUS-GJ for duodenal obstruction is reserved for patients not deemed candidates for surgical resection. Herein, we describe employing EUS-GJ using LAMS as a salvage procedure in a patient with an obstructed duodenal stent during neoadjuvant treatment for DAC that ultimately proceeded to surgical resection by a classic pancreaticoduodenectomy (PD).

## Case report

**Fig. 1:**
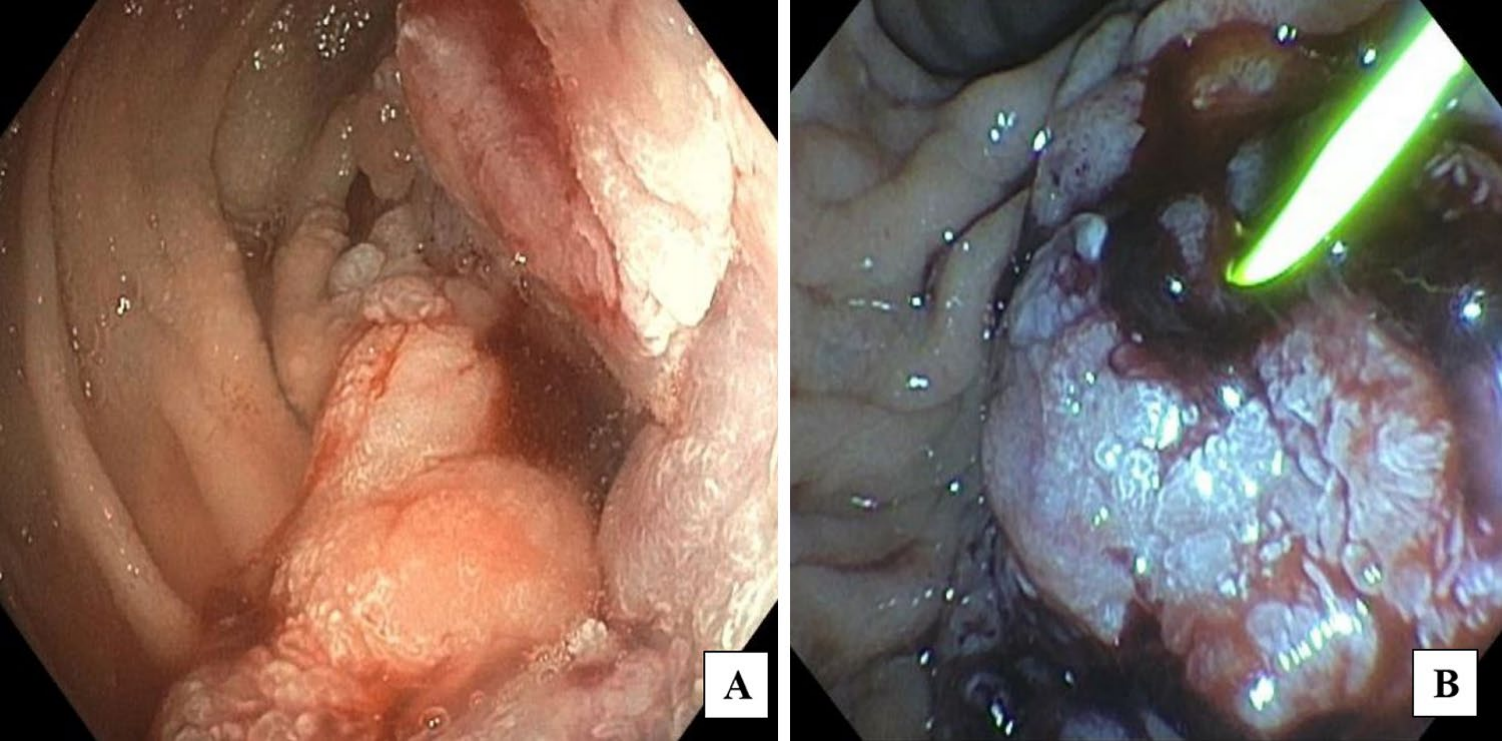
Endoscopic images of duodenal mass and ampulla involvement of the mass. **A** Duodenal mass with narrowing of the duodenal lumen. **B** Biliary obstruction at the level of the ampulla

**Fig. 2:**
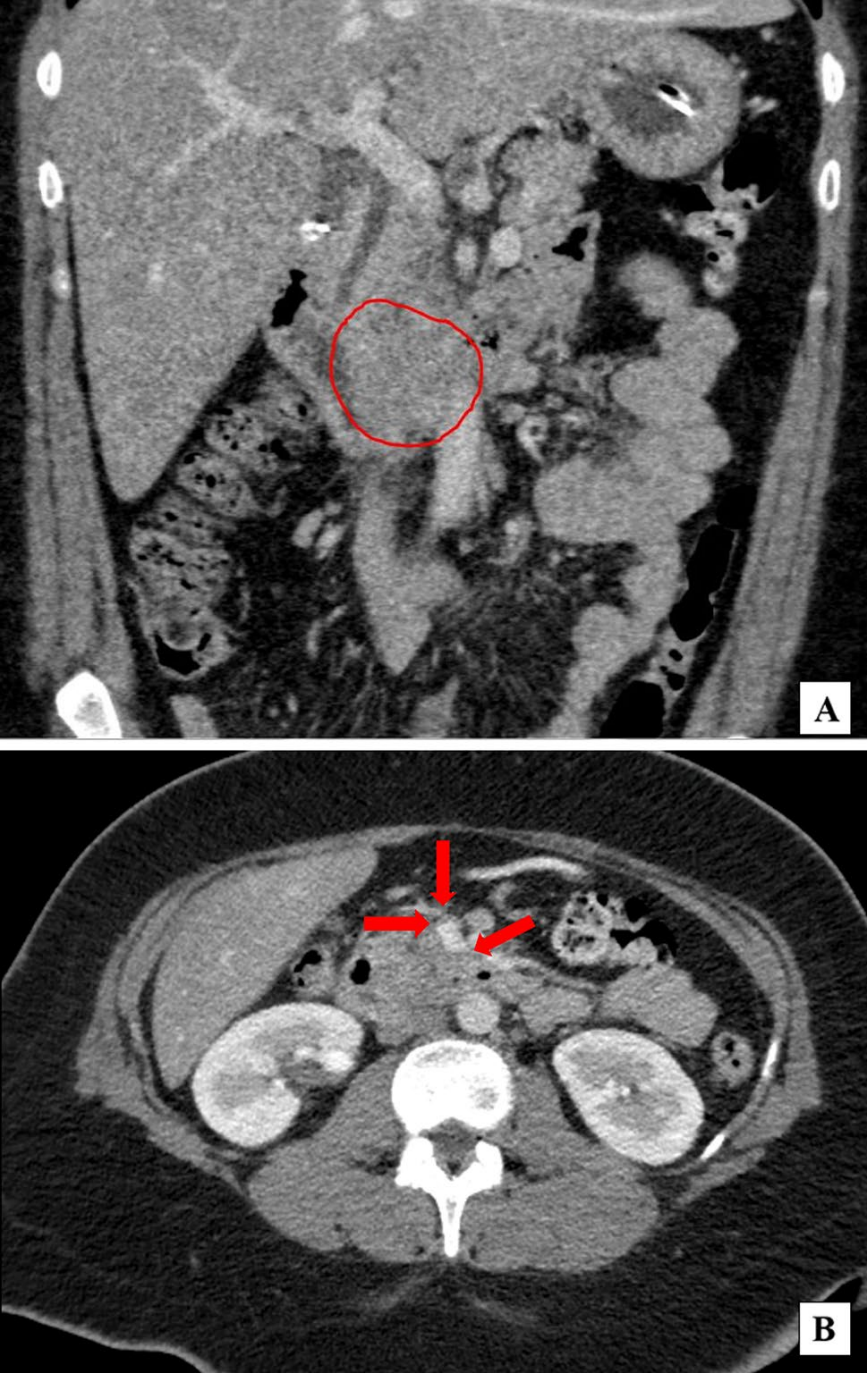
Coronal and axial abdominal CT images with intravenous contrast demonstrating duodenal mass and adjacent vessels. **A** Red circle encompasses 4.5 cm × 2.6 cm mass in the proximal third portion of the duodenum at the inferior duodenal flexure causing duodenal narrowing and biliary obstruction. **B** Red arrows point to locally advanced tumor, which is abutting the superior mesenteric vessels. The vessels remain patent

**Fig. 3:**
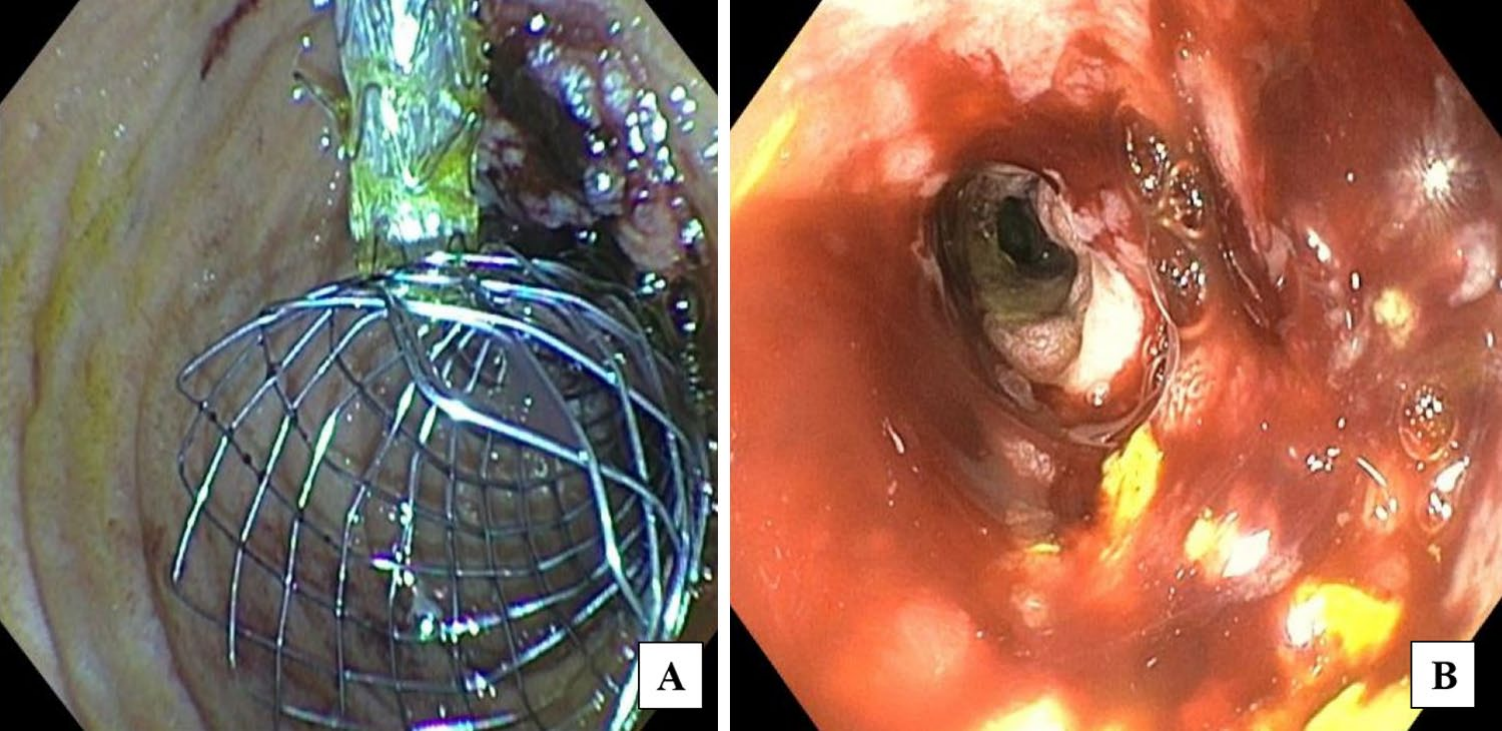
Endoscopic image of common bile duct and duodenal stents. **A** Endoscopic image of common bile duct (CBD) 8 mm × 60 mm self-expanding metal stent (FCSEMS, Viabil) and duodenal 22 mm × 90 mm uncovered metal stent (USEMS, Wallflex). **B** Repeat endoscopy after patient presented with nausea and vomiting revealed duodenal stent stenosis with tumor ingrowth

**Fig. 4:**
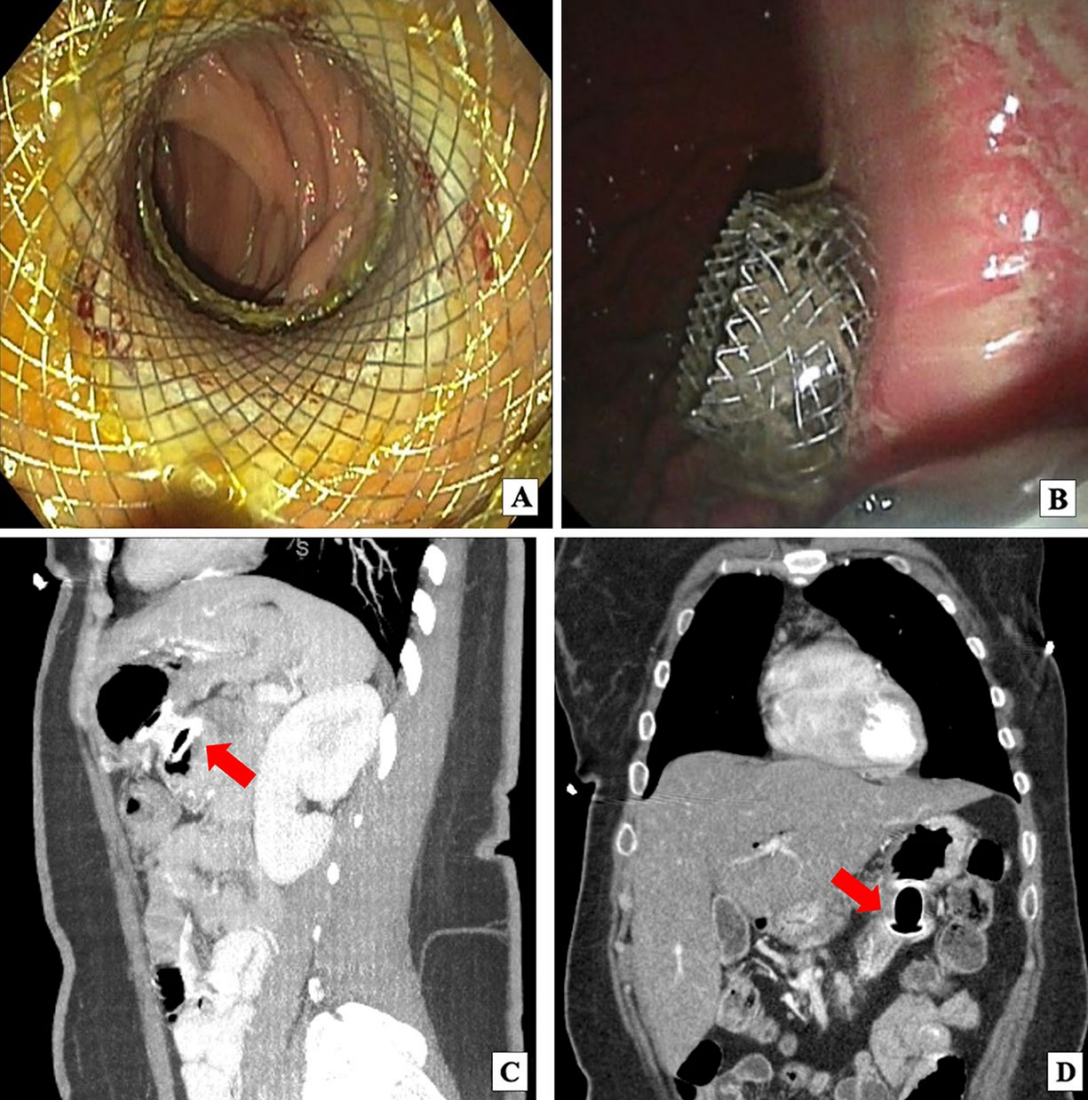
Endoscopic images of lumen apposing metal stent (LAMS) deployment between stomach and jejunum and abdominal CT images with intravenous contrast demonstrating LAMS gastrojejunostomy (GJ). **A** Jejunum as seen through the LAMS. After contrast was injected to identify the jejunal lumen distal to the duodenal stent that had become stenosed, a 20 mm × 10 mm electrocautery-enhanced LAMS (Hot Axios, BSCI) was deployed from the stomach to the small bowel just distal to the duodenal stent. The LAMS was dilated to 18 mm using a wire-guided balloon dilator. Small bowel was examined distal to the LAMS which was healthy. No trauma from the LAMS was demonstrated. **B** Deployment of LAMS flange in the stomach. The distance between the gastric and jejunal wall was less than 1 cm. There were no significant blood vessels in the path chosen for LAMS entry. **C** Sagittal abdominal CT with red arrow pointing to interval LAMS placed between posterior gastric wall and proximal jejunum, distal to the end of the obstructed duodenal stent. **D** Coronal abdominal CT with red arrow pointing to LAMS between stomach and proximal jejunum

**Fig. 5:**
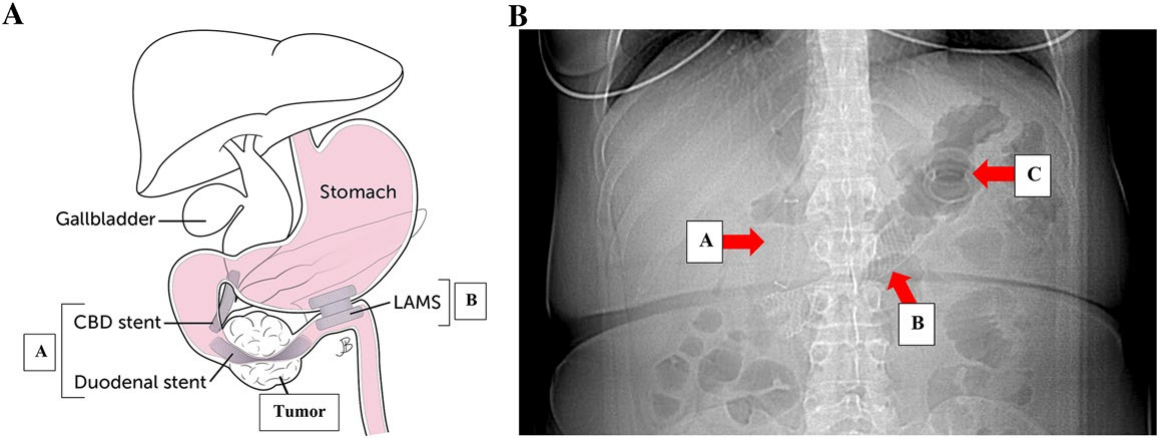
**A** Location of common bile duct (CBD), duodenal, and GJ stents relative to duodenal mass. **A** Cannulation of the bile duct, biliary sphincterotomy, and 8 mm × 60 mm fully covered self-expanding metal stent (FCSEMS, Viabil Fore) placement into biliary duct with distal end in duodenum were performed via ERCP. Circumferential, fungating, obstructing mass in the distal descending duodenum involving the major papilla causing 3 cm stricture was traversed with pediatric colonoscope under endoscopic and fluoroscopic guidance. **A** 22 mm × 90 mm uncovered self-expanding metal stent (USEMS, WallFlex BSCI) was placed across the duodenal stricture, with the proximal end at the level of the major papilla near the previously placed biliary stent. **B** LAMS was placed two and a half months later. **B** Abdominal X-ray with red arrows pointing to CBD (**A**), duodenal (**B**), and LAM (**C**) stents

A 51-year-old woman presented with abdominal fullness, nausea, vomiting, and weight loss. Abdominal imaging revealed a 4.5 × 2.6 cm distal duodenal mass with narrowing of the duodenal lumen and biliary obstruction at the level of the ampulla (Fig. 1), abutment of superior mesenteric vessels, and portacaval lymphadenopathy consistent with locally advanced disease (Fig. 2). EUS fine-needle biopsy demonstrated moderately differentiated adenocarcinoma. A common bile duct (CBD) 8 mm × 60 mm self-expanding metal stent (FCSEMS, Viabil) and duodenal 22 mm × 90 mm uncovered metal stent (USEMS, Wallflex) were placed (Fig. 3A). In the interim, the patient began neoadjuvant FOLFOX (folinic acid, fluorouracil, oxaliplatin) and completed three cycles. Two and a half months after CBD and duodenal stent placement, the patient presented with nausea and emesis. Repeat endoscopy revealed duodenal stent stenosis with tumor ingrowth (Fig. 3B). EUS-GJ was subsequently performed using a 20 mm LAMS (Axios, BSCI) from the distal gastric body to the proximal jejunum (Figs. 4, 5A,B). All three stents—CBD, duodenal, and LAMS—are depicted in Fig. 5A,B. Given the short time interval after the initial duodenal stent placement, a second duodenal stent was determined unable to provide durable endoluminal patency for adequate nutritional support prior to surgery. In our patient, following EUS-GJ using LAMS, the patient tolerated liquids the same day and stent diet within 48 h. She resumed systemic neoadjuvant therapy and completed three additional cycles prior to surgical resection 14 weeks after LAMS placement.

Surgical resection of the duodenal mass required a classic pancreaticoduodenectomy (PD)/Whipple procedure. The previously placed LAMS traversed the mesocolon, creating significant inflammation and fibrosis. This required en bloc resection of the stent along with the gastric body and proximal jejunum. The patient tolerated the procedure well and was discharged on post-operative day 5 [3]. Four days following discharge, the patient required hospital readmission for confirmed post-operative pancreatic fistula (POPF) requiring percutaneous drainage with resultant resolution of the fistula. The POPF was a suspected complication of the surgery and wound healing, plausibly attributed to the GJ-LAMS. Pathology on the Whipple specimen revealed a grade II duodenal adenocarcinoma (pT3N1), with 2/16 specimen lymph nodes positive for metastatic adenocarcinoma and negative margins. The patient subsequently completed six cycles of adjuvant FOLFOX chemotherapy and currently is without evidence of disease recurrence.

## Discussion

This case report highlights EUS-GJ using LAMS during the neoadjuvant setting as an effective strategy or bridge to definitive surgery in a patient with an obstructed duodenal stent from disease progression. The advent of LAMS significantly changed the landscape of therapeutic endoscopy. LAMS, first described by Binmoeller and Shah in 2011 [4], has a few variations commercially available, but universally contains a barbell or saddle configuration allowing apposition of two lumens or cavities. Transluminal placement of LAMS requires EUS. Initially developed to facilitate endoscopic drainage of peripancreatic fluid collections, indications for LAMS continue to expand, including off-label use for EUS-GJ in the palliative setting [5, 6].

The EUS-GJ in our patient was performed using the following standard technique that is used for all of our patients at our institution. A guidewire was advanced through the duodenal stricture into the proximal jejunum. A nasojejunal tube was then advanced over the guidewire and left in the proximal jejunum close to the ligament of Treitz. The proximal jejunum was distended with diluted contrast and identified on fluoroscopy. With echoendoscope in the stomach, the proximal jejunum being actively and adequately distended was identified and a 20 mm LAMS was placed free hand creating a successful endoscopic gastrojejunostomy.

Krishnamoorthi and colleagues, in a recent systematic review and meta-analysis, compared EUS-GJ, duodenal stent, and surgical gastrojejunostomy. They report a technical success rate of 95.3% and favorable clinical outcome in 89% of patients with EUS-GJ [2]. EUS-GJ with LAMS provided a durable stent patency compared to duodenal stent (re-intervention rate of 11.3% vs 20.3%, respectively) for palliation of malignant gastric outlet obstruction (GOO) [2]. Neoadjuvant treatment with EUS-GJ was an effective management strategy for our patient with duodenal stent stenosis from tumor growth. Notably, adverse events with EUS-GJ include bleeding (2.9%), perforation (2.8%), stent migration (2.4%), and stent occlusion (0.5%) [2].

In our patient, EUS-GJ for an obstructing duodenal adenocarcinoma after a failed duodenal stent did not preclude safe surgical resection. Indeed, EUS-GJ with LAMS can potentially be an effective salvage strategy in highly select patients. LAMS successfully bridged our patient during 14 weeks to surgical resection. However, use of LAMS may accentuate inherent risks and challenges previously reported for stents in the perioperative period [7–9]. From a surgical standpoint, inclusion of the LAMS gastric site within the surgical specimen necessitated performance of a classic PD rather than our standard pylorus-preserving PD (PPPD) [10]. Moreover, significant inflammation, fibrosis, potential for field contamination, and added technical complexity can predispose patients to potential procedure-related risks, surgical site infection (SSI), and morbidity.

Notably, use of EUS-GJ for duodenal obstruction from pancreatic cancer followed by pancreaticoduodenectomy was reported in a single case by a group from Italy [11]. The same investigators also described surgical feasibility after EUS-GJ in a review [12]. Currently, EUS-GJ in the United States is largely considered a palliative measure and not utilized for patients deemed potential candidates for surgical resection, as described in our patient with locally advanced DAC.

The limitations of this case report include its retrospective nature, lack of ability to generalize to a larger sample, and inability to establish a cause-and-effect relationship.

With the expanded indications for LAMS and its ease of use, gastroenterologists and surgeons will encounter opportunities for using LAMS more frequently in the future for a variety of pathologies. Clinical considerations utilizing EUS-GJ with LAMS as a potential bridge to surgical resection warrant further investigation. However, the potential for durable restoration of intestinal continuity in the neoadjuvant setting makes EUS-GJ with LAMS an attractive option. Ultimately, the decision on the appropriate modality for management and treatment rests upon multidisciplinary review and patient involvement during the shared decision-making process.
